# Pneumomediastinum in Acute Asthma Exacerbation

**DOI:** 10.7759/cureus.94887

**Published:** 2025-10-18

**Authors:** Amrit S Banga, Simran S Banga, Francis J Asino, Sivakumar Nagaraju

**Affiliations:** 1 Emergency Medicine, Touro University California College of Osteopathic Medicine, Vallejo, USA; 2 Anesthesiology, Naval Medical Center San Diego, San Diego, USA; 3 Internal Medicine, Touro University California College of Osteopathic Medicine, Vallejo, USA; 4 Critical Care Medicine, Burrell College of Osteopathic Medicine, Las Cruces, USA; 5 Pulmonary and Critical Care Medicine, Lovelace Medical Center, Albuquerque, USA

**Keywords:** acute asthma exacerbation, acute asthma exacerbation management, acute hypoxemic respiratory failure, air leak syndrome, anterior mediastinum, asthma, hypoxia, mediastinal emphysema, pneumomediastinum, subcutaneous emphysema

## Abstract

Pneumomediastinum (PM) is a rare but significant complication of asthma exacerbation. We present the case of a 57-year-old woman with a history of asthma and alcoholic liver cirrhosis who presented to the ED with severe shortness of breath and an oxygen saturation of 83% on room air, as reported by EMS. On arrival, she was tachycardic and hypoxic, requiring 5 L/min of oxygen via nebulizer mask, with diffuse wheezing noted on auscultation. Despite aggressive medical management, including high-flow oxygen, bronchodilators, and steroids, her symptoms persisted. A chest radiograph was unremarkable; however, a CT scan of the chest revealed air tracking along the anterior mediastinum without evidence of pneumothorax. Given her clinical stability and the absence of esophageal injury on fluoroscopy, she was managed conservatively with close observation. Repeat imaging demonstrated resolution of the PM without surgical intervention. The patient was hospitalized for 19 days and discharged in stable condition with normal vital signs, with outpatient follow-up arranged with her primary care physician and pulmonologist. This case highlights the importance of considering PM in the differential diagnosis of patients with refractory asthma exacerbations, particularly in older adults, despite its more frequent association with pediatric and trauma populations. Early imaging in non-responding asthma patients may facilitate timely diagnosis and appropriate management.

## Introduction

Pneumomediastinum (PM) is a rare condition characterized by the presence of free air within the mediastinum. Its pathophysiology is explained by the Macklin effect, in which increased intrathoracic pressure causes alveolar rupture, allowing air to track along the bronchovascular sheaths into the mediastinum [1,2]. While PM is commonly associated with trauma, foreign body aspiration, or vigorous physical activity, spontaneous PM is an uncommon complication of asthma exacerbation and is typically identified as an abnormal finding on imaging such as chest radiography or CT [3,4].

The true incidence of PM in asthma remains largely unknown and is likely underdiagnosed due to its nonspecific symptoms, which may include chest pain, dyspnea, and subcutaneous emphysema [5]. In most cases, PM resolves spontaneously without the need for invasive intervention [5]. However, failure to recognize this condition can result in unnecessary diagnostic workups or missed identification of other serious complications, such as pneumothorax or esophageal perforation [3].

Clinicians rely on imaging modalities, including chest radiography and CT, to confirm the diagnosis [6]. Depending on the extent of the PM, physical examination findings may include crepitus or a crunching sound that can be auscultated or palpated over the chest wall [7].

This case report describes an unusual presentation of an asthma exacerbation in a 57-year-old woman with a medical history of asthma and alcoholic liver cirrhosis. Her case underscores the diagnostic challenges of identifying PM in older adults without classic risk factors and highlights the importance of early imaging in refractory asthma. Additionally, her oxygenation may have been more difficult to maintain not only because of asthma and PM but also due to impaired baseline pulmonary reserve from cirrhosis, which can limit effective diaphragmatic contraction.

## Case presentation

A 57-year-old woman with a past medical history of asthma and alcoholic liver cirrhosis presented to the ED with a one-day history of worsening shortness of breath and cough. She reported chest tightness and difficulty breathing but denied chest pain. Home use of albuterol inhalers and nebulizers provided no relief.

**Figure 1:**
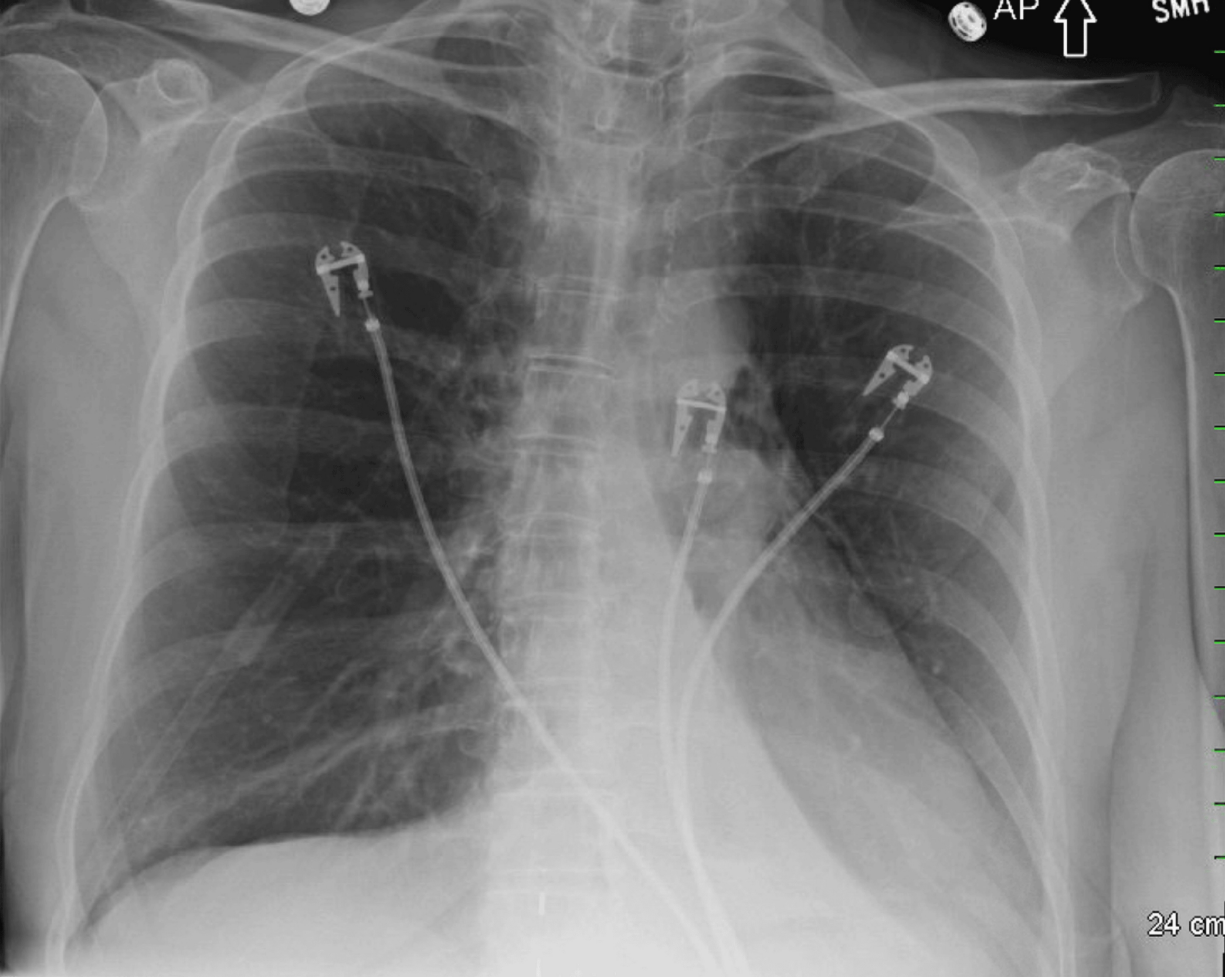
Portable chest radiograph on admission showing no evidence of PM PM, pneumomediastinum

Emergency medical services noted an oxygen saturation of 83% on room air, prompting ambulance transport to the ED. On arrival, her vital signs showed a heart rate of 102 beats per minute and an oxygen saturation of 97% on 5 L/min of oxygen via nebulizer mask. Physical examination revealed bilateral wheezing on auscultation. Laboratory results demonstrated eosinophilia at 8.4%, consistent with an acute inflammatory process. Troponin was negative, and her comprehensive metabolic panel was within normal limits (Table 1). Portable chest radiography was unremarkable (Figure 1).

**Table 1:** Laboratory results on admission and at discharge BUN, blood urea nitrogen; GFR, glomerular filtration rate; MCV, mean corpuscular volume; MCHC, mean corpuscular hemoglobin concentration; RDW, red cell distribution width

Lab value	Admission	Discharge	Reference range
WBC	10.4	12.5	4.5-11.0 × 10³/µL
RBC	4.45	3.81	3.8-5.2 × 10⁶/µL
Hemoglobin	14.4	12.6	12.0-15.5 g/dL
Hematocrit	42.1	36.3	36-44%
MCV	94.6	95.3	80-100 fL
MCHC	34.2	34.7	32-36 g/dL
RDW	15	14.9	11.5-14.5%
Sodium	133	135	135-145 mmol/L
Potassium	5.1	4	3.5-5.0 mmol/L
Chloride	103	104	98-106 mmol/L
CO₂	23	23	22-29 mmol/L
BUN	32	20	7-20 mg/dL
Creatinine	0.8	0.6	0.6-1.1 mg/dL
Calcium	8.4	8.3	8.5-10.5 mg/dL
Estimated GFR	85	105	>60 mL/min/1.73 m²
BUN/creatinine ratio	40	33	10:1-20:1
Anion gap	7	6	8-12 mEq/L

The patient was admitted for acute hypoxic respiratory failure due to a persistent oxygen requirement of 5 L/min via nebulizer mask, which deviated from her baseline, as she did not require supplemental oxygen at home. Given concern for asthma exacerbation, she was treated with nebulized albuterol for two days and intravenous methylprednisolone 40 mg every 12 hours for six days. Additionally, nebulized acetylcysteine was administered for three days for suspected mucus plugging in the setting of airway obstruction and hypoxemia.

**Figure 2:**
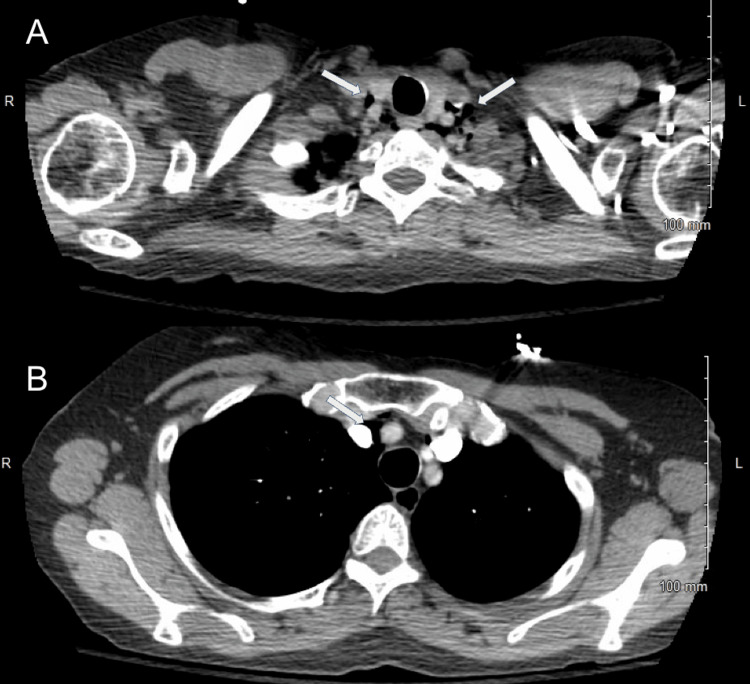
CT of the chest demonstrating PM (A) Superior axial slice showing air in the anterior mediastinum. (B) Inferior axial slice also demonstrating anterior mediastinal air without pneumothorax. PM, pneumomediastinum

**Figure 3:**
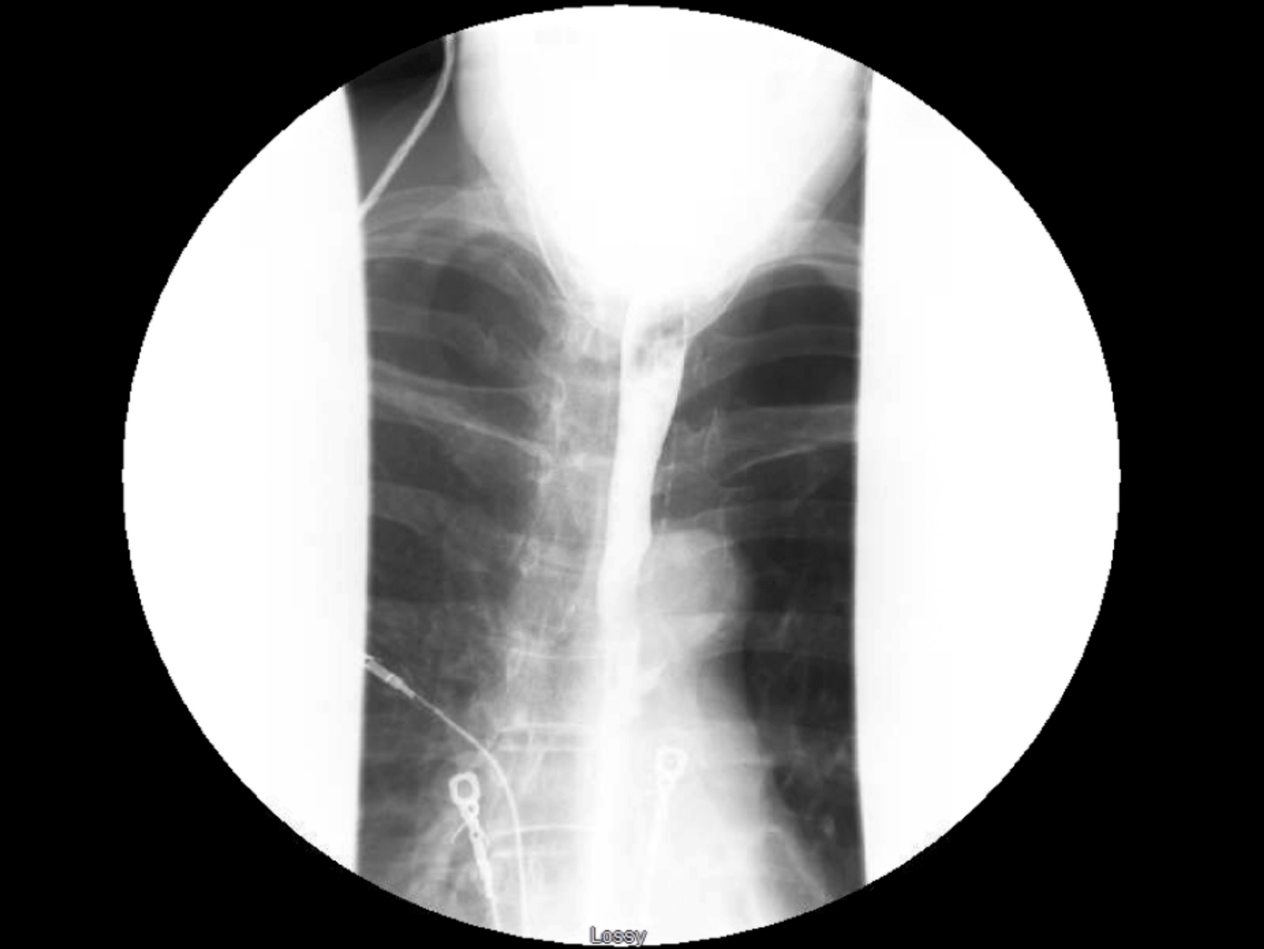
Fluoroscopic esophagram showing no evidence of esophageal perforation

Despite therapy, her oxygen saturation did not improve, and she continued to experience dyspnea. On hospital day 4, a chest CT with pulmonary embolism protocol was performed, revealing pockets of air in the anterior mediastinum, consistent with PM (Figure 2). A subsequent fluoroscopic esophagram showed no evidence of esophageal perforation or air leak (Figure 3).

**Figure 4:**
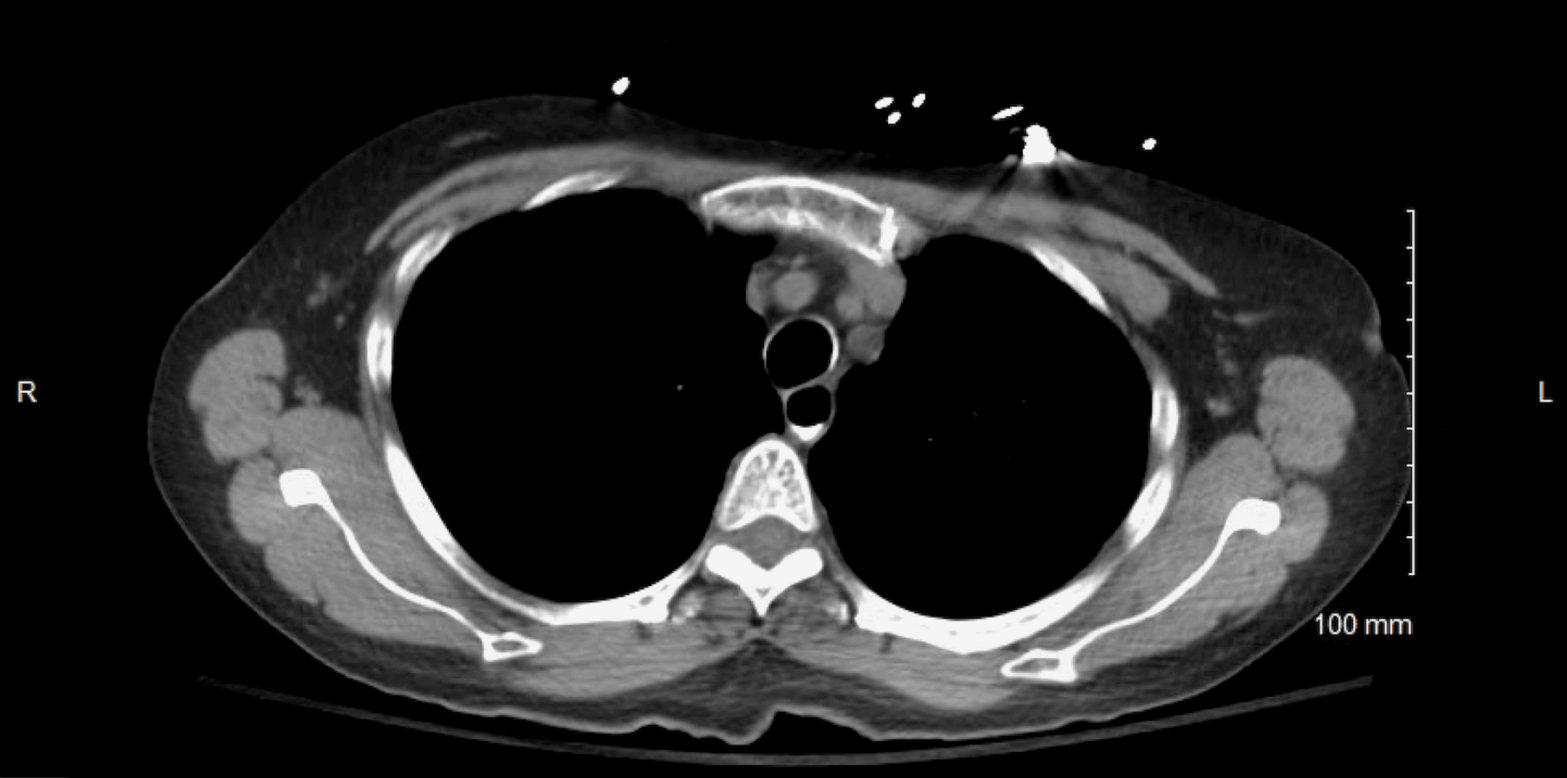
Repeat CT of the chest demonstrating resolution of PM PM, pneumomediastinum

**Figure 5:**
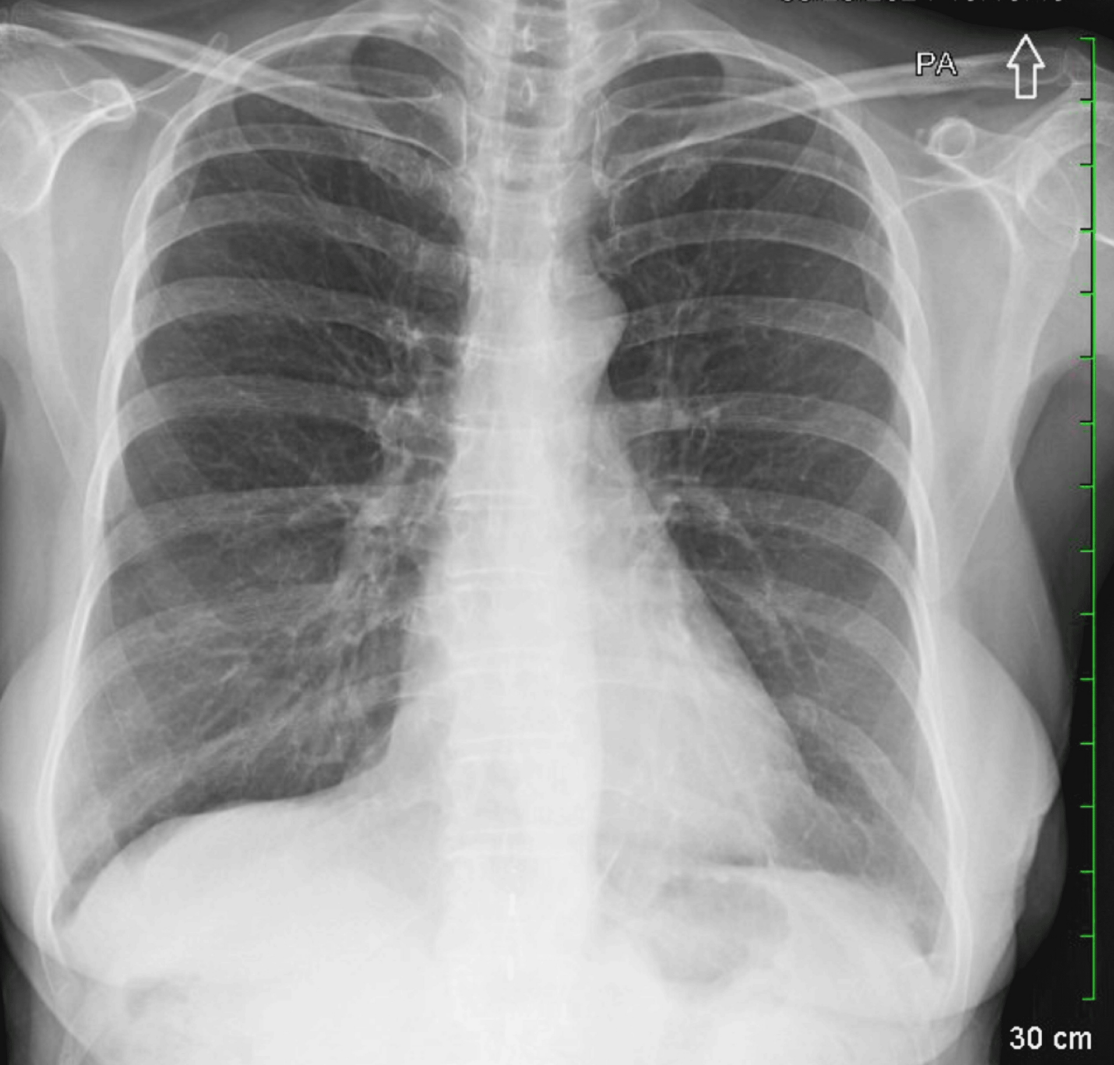
Repeat chest radiograph at discharge showing no evidence of PM PM, pneumomediastinum

Given her clinical stability and absence of complications, she was managed conservatively with oxygen support and close observation. Repeat imaging confirmed complete resolution of the PM without the need for surgical or thoracic intervention (Figure 4, Figure 5). After a 19-day hospitalization, she was stable on room air. Her discharge laboratory results were notable for leukocytosis, likely steroid-induced, but were otherwise unremarkable. She was discharged on a prednisone taper starting at 40 mg daily for three weeks, with outpatient follow-up arranged with her primary care physician and pulmonologist.

## Discussion

In contrast to previously reported cases, such as a 63-year-old woman with PM [8] and younger patients with identifiable triggers [9-11], our patient had no history of exertion or trauma. Reported triggers in other cases include blunt force trauma and excessive physical exertion, though PM can also occur spontaneously. The air detected in the anterior mediastinum was most likely caused by excessive coughing, leading to alveolar rupture in the upper lung apices and subsequent air leakage into the mediastinum. Crepitus, often cited as a physical examination finding associated with PM [7], was absent in our patient, underscoring the importance of maintaining a low threshold for imaging in cases of persistent dyspnea or hypoxia.

A review of the literature shows that PM secondary to asthma exacerbation is most commonly reported in the pediatric population, particularly in those with foreign body aspiration [9]. Other reports describe PM in athletes after vigorous exercise, such as cycling [10], or following blunt trauma during contact sports [11]. Additional cases involve PM associated with isolated blunt chest trauma [11]. Our patient had no such precipitating factors, which initially lowered the suspicion of PM in the differential diagnosis.

The incidence of PM in asthma exacerbations remains uncertain [5]. In one study of 45 adults admitted for acute asthma exacerbation, chest radiography and arterial oxygen saturation (SpO₂) monitoring were performed 24 hours after admission. Patients with persistent or worsening hypoxia underwent chest CT, and PM was diagnosed in five of the 45 patients [5]. That study noted PM occurred more frequently in younger patients but did not report gender distribution [5]. In contrast, our patient was 57 years old. Another study of 100 patients with primary spontaneous PM found that only 3% were older than 50 years [3], highlighting the rarity of PM in older adults. This reinforces the importance of considering PM in the differential diagnosis regardless of age.

The clinical presentation of PM is variable, but retrospective data help contextualize our patient’s findings. The most common presenting symptoms are chest pain (76%), dyspnea (57%), and neck pain (47%), with subcutaneous emphysema (33%) being the most frequent physical finding [3]. Tachycardia occurs in about 31% of cases, and hypoxia is uncommon [3]. Our patient reported vague chest pressure and discomfort during the first 48 hours of hospitalization; however, unlike typical cases, she presented with hypoxia (SpO₂ 83% on room air) and tachycardia (102 bpm). While her asthma and recent cough were consistent with previously reported associations, her age and comorbid alcoholic cirrhosis deviated from the typical demographic. Notably, we did not find any literature establishing a direct causal link between liver cirrhosis and spontaneous PM.

Chest radiography is the first-line imaging modality in suspected PM and is diagnostic in approximately 69% of cases, though it may miss early or small-volume mediastinal air [2,4,5,12]. When chest radiography is inconclusive or clinical deterioration persists, CT imaging is recommended to confirm the diagnosis and assess the extent of mediastinal air, as well as to rule out other thoracic pathologies. In a case series, CT confirmed PM in 80% of cases where chest radiography was inconclusive [4,5,12,13]. In acute asthma exacerbations, comorbidities such as chronic obstructive pulmonary disease, heart failure, or prior lung infections may affect imaging sensitivity [6,13]. In our case, the initial chest radiograph was unremarkable, but subsequent CT confirmed the presence of mediastinal air, emphasizing the value of advanced imaging when clinical suspicion remains high.

PM can often be managed successfully with conservative measures, even in severe presentations. However, clinicians should remain vigilant for deterioration, especially when atypical features are present [4]. Management is typically supportive, including bed rest, analgesia, and supplemental oxygen [14]. Supplemental oxygen accelerates mediastinal air reabsorption by increasing the diffusion gradient for nitrogen. While earlier reports supported dietary restriction and prophylactic antibiotics to prevent mediastinitis [15,16], more recent evidence shows limited benefit in low-risk patients, those younger than 40 years, with normal white blood cell counts, no pleural effusion, and low suspicion of esophageal perforation. For such patients, advancing diet as tolerated and avoiding prophylactic antibiotics is generally recommended [3,14].

Our patient, however, was 57 years old, presented with persistent hypoxia, and had abnormal imaging findings. These higher-risk features warranted prolonged inpatient monitoring, conservative management with supplemental oxygen, and repeat imaging until resolution, without the need for antibiotic prophylaxis.

Although rare, complications of PM include pneumothorax, airway compression, tension pneumopericardium, or tracheobronchial injury, which may necessitate invasive interventions such as thoracostomy, thoracoscopy, or pericardial window placement [14]. None of these complications occurred in our patient, who improved with conservative management and supportive care alone.

## Conclusions

This case highlights the importance of including PM in the differential diagnosis of patients with acute asthma exacerbations who fail to improve despite aggressive treatment. Although PM is more frequently reported in younger populations, this case demonstrates that it can also occur in older adults and may present with refractory hypoxia despite appropriate management. Awareness of potential complications underscores the importance of cautious inpatient monitoring, while early recognition through imaging and timely supportive care remain central to achieving favorable outcomes.

## References

[1] Klamfoth J, Koroscil M (2024). Spontaneous pneumomediastinum secondary to undiagnosed asthma in military adult. Mil Med.

[2] Romero KJ, Trujillo MH (2010). Spontaneous pneumomediastinum and subcutaneous emphysema in asthma exacerbation: the Macklin effect. Heart Lung.

[3] Morgan CT, Kanne JP, Lewis EE, Maloney JD, DeCamp MM, McCarthy DP (2023). One hundred cases of primary spontaneous pneumomediastinum: leukocytosis is common, pleural effusions and age over 40 are rare. J Thorac Dis.

[4] Caceres M, Ali SZ, Braud R, Weiman D, Garrett HE Jr (2008). Spontaneous pneumomediastinum: a comparative study and review of the literature. Ann Thorac Surg.

[5] Vianello A, Caminati M, Chieco-Bianchi F (2018). Spontaneous pneumomediastinum complicating severe acute asthma exacerbation in adult patients. J Asthma.

[6] Batra K, Walker CM, Little BP (2025). ACR Appropriateness Criteria® Acute Respiratory Illness in Immunocompetent Patients: 2024 update. J Am Coll Radiol.

[7] Kardon EM (1996). Acute asthma. Emerg Med Clin North Am.

[8] Kogan I, Celli BR (2000). Pneumomediastinum in a 63-year-old woman with asthma exacerbation. Chest.

[9] Bourrous M, Lahmini W, Nouri H, Haimeur N (2019). Subcutaneous emphysema and pneumomediastinum in child with asthma revealing occult foreign body aspiration: a case report. J Med Case Rep.

[10] Zafar F, Afzal O (2023). Chest tightness with cycling turned out to be pneumomediastinum. Am J Case Rep.

[11] Vanzo V, Bugin S, Snijders D, Bottecchia L, Storer V, Barbato A (2013). Pneumomediastinum and pneumopericardium in an 11-year-old rugby player: a case report. J Athl Train.

[12] Fan MI, Goh S, Choi J, Tan DJ (2024). Spontaneous pneumomediastinum and pneumopericardium in a young male with asthma. J Asthma.

[13] Jokerst C, Chung JH, Ackman JB (2018). ACR Appropriateness Criteria(®) Acute Respiratory Illness in Immunocompetent Patients. J Am Coll Radiol.

[14] Susai CJ, Banks KC, Alcasid NJ, Velotta JB (2024). A clinical review of spontaneous pneumomediastinum. Mediastinum.

[15] Jougon JB, Ballester M, Delcambre F, Mac Bride T, Dromer CE, Velly JF (2003). Assessment of spontaneous pneumomediastinum: experience with 12 patients. Ann Thorac Surg.

[16] Koullias GJ, Korkolis DP, Wang XJ, Hammond GL (2004). Current assessment and management of spontaneous pneumomediastinum: experience in 24 adult patients. Eur J Cardiothorac Surg.

